# Stabilizing stretch reflexes are modulated independently from the rapid release of perturbation-triggered motor plans

**DOI:** 10.1038/s41598-019-50460-1

**Published:** 2019-09-26

**Authors:** Hyunglae Lee, Eric J. Perreault

**Affiliations:** 1 School for Engineering of Matter, Transport, and Energy, Arizona State University, Tempe, AZ 85287 USA; 2 Department of Biomedical Engineering, Northwestern University, Evanston, IL 60208 USA; 3 Department of Physical Medicine and Rehabilitation, Northwestern University, Chicago, IL 60611 USA; 4 Shirley Ryan Ability Lab, Chicago, IL 60611 USA

**Keywords:** Neuromuscular junction, Motor cortex

## Abstract

Responses elicited after the shortest latency spinal reflexes but prior to the onset of voluntary activity can display sophistication beyond a stereotypical reflex. Two distinct behaviors have been identified for these rapid motor responses, often called long-latency reflexes. The first is to maintain limb stability by opposing external perturbations. The second is to quickly release motor actions planned prior to the disturbance, often called a triggered reaction. This study investigated their interaction when motor tasks involve both limb stabilization and motor planning. We used a robotic manipulator to change the stability of the haptic environment during 2D arm reaching tasks, and to apply perturbations that could elicit rapid motor responses. Stabilizing reflexes were modulated by the orientation of the haptic environment (field effect) whereas triggered reactions were modulated by the target to which subjects were instructed to reach (target effect). We observed that there were no significant interactions between the target and field effects in the early (50–75 ms) portion of the long-latency reflex, indicating that these components of the rapid motor response are initially controlled independently. There were small but significant interactions for two of the six relevant muscles in the later portion (75–100 ms) of the reflex response. In addition, the target effect was influenced by the direction of the perturbation used to elicit the motor response, indicating a later feedback correction in addition to the early component of the triggered reaction. Together, these results demonstrate how distinct components of the long-latency reflex can work independently and together to generate sophisticated rapid motor responses that integrate planning with reaction to uncertain conditions.

## Introduction

Controlling arm posture and generating purposeful movements requires appropriate planning and execution of the neural commands responsible for eliciting a movement and responding to unanticipated postural disturbances. Once the commands for a desired motor action are learned, even complicated tasks can be achieved with remarkably little cognitive burden, suggesting a significant reliance on involuntary motor actions. These responses help us execute rapid movements, respond to external stimuli, and correct for errors in posture and movement control. Given that many functional tasks require the ability to plan for desired movements, and stable movement execution in the presence of environmental uncertainty, it is important to investigate how these abilities are integrated into the most rapid motor responses.

The stretch reflex, broadly defined as a rapid motor response to external perturbations of posture, is an integral component of the involuntary motor system. Different components of the stretch reflex have been reported. The earliest involuntary muscle responses to perturbations of the upper limb occur approximately 20 ms after perturbation onset for biceps brachii^1^, and have been known to contribute to the regulation of muscle stiffness^2,3^ and joint stiffness^4^. This short-latency reflex (SLR) is associated with a spinal monosynaptic pathway^5,6^, distinctive from the earliest voluntary muscle response which occurs 90–100 ms following the onset of stimulus^7^. While many previous studies have been separated along these lines, it is now widely recognized that a strict dichotomy does not exist between involuntary and voluntary control^8^. Rather, there is a continuum within which lies the long-latency reflex (LLR), a response rapid enough to be considered reflexive, yet adaptable in a way that reflects volitional control^9^.

Distinct functional roles of the LLR have been reported. Stabilizing reflexes to maintain limb stability are modulated in a task appropriate manner in response to the mechanics between the upper limb and the environment; interactions with compliant or destabilizing environments increase stretch reflex sensitivity^10–14^. Further, stabilizing reflexes are modulated in a manner consistent with having an internal model of limb dynamics, suggesting that LLR share many of the functional properties of voluntary control^15–17^.

Long-latency reflexes also contribute to hasten and augment the planned motor actions of the upper limb. In the presence of a movement plan, a brief mechanical perturbation induces the early release of planned movement, a phenomenon often called triggered reaction^18–21^. This response decreases movement time substantially, to within the range attributed to the LLR and much faster than voluntary movement. Modulation of a triggered reaction can occur to account for changing requirements of a task, such as a moving target, instructions provided to a subject, or as a reflection of an underlying decision-making process, all likely indicative of feedback control after movement initiation^17,22–24^.

While distinct functional roles of the LLR have been investigated, little is known about how they interact during tasks simultaneously requiring the regulation of stability and rapid execution of planned motor actions. The objective of this study was to investigate these interactions. We used directional haptic environments to preferentially tune stabilizing reflexes independently from the muscle activity required for planned movements. Based upon growing evidence of the multiple convergent pathways contributing to LLR^16,25–28^, we hypothesized that stabilizing reflexes and rapidly-released planned motor responses can be modulated independently. We further explored when these responses can be modulated by feedback control. Our results demonstrate how the multiple convergent pathways contributing to LLR can work independently and in coordination to generate sophisticated rapid motor responses that integrate planning with reaction to uncertain conditions.

## Materials and Methods

### Participants

Ten right-handed subjects (23–34 yr., 5 males and 5 females) with no reported history of neurological disorders or orthopedic limitations in the upper limbs were recruited for this study. All protocols were approved by the Northwestern University Institutional Review Board (IRB protocol STU00009204). Subjects provided informed, written consent prior to participation. All experimental procedures were performed in accordance with the relevant guidelines and regulations.

### Equipment

Details of the equipment have been provided previously^13^. In summary, a three degrees-of-freedom (DOF) robotic manipulator (Haptic Master; Moog FCS, Nieuw-Vennep, The Netherlands) was used to change the stability of the environment during 2D arm reaching tasks and to apply perturbations that could elicit stabilizing stretch reflexes and rapid, target-dependent reactions.

The robot was used in an admittance control mode to simulate a range of virtual environments^29^. When perturbations were applied, the robot was transiently switched to a stiff position servo mode (50 kN/m) so that perturbation kinematics could be controlled precisely. The switching time between the admittance controller and the position servo was less than 1 ms^13^. Subjects were seated with their trunk securely strapped to a rigid chair, facing a visual display at a distance of ~1 m. The arm was securely attached to the robot using a custom-fitted fiberglass cast mounted to a gimbal at the end of the manipulator. Potentiometers embedded in the gimbal provided subjects with visual feedback of arm orientation to help maintain a target arm posture at the beginning of each experimental trial. This posture positioned the hand in front of the glenohumeral joint with the shoulder in ~70° of abduction, ~45° of horizontal flexion, and the elbow in ~90° of flexion. This starting posture was considered the “home position” for all trials.

Surface electromyograms (EMGs) were recorded from eight muscles that span the shoulder and elbow joints: brachioradialis (BRD), biceps brachii (BI), long head of triceps brachii (TRI_LONG_), lateral head of triceps brachii (TRI_LAT_), clavicular head of pectoralis major (PECT_CLAV_), anterior deltoid (AD), middle deltoid (MD), and posterior deltoid (PD). In addition, the left and right sternocleidomastoid (SCM) muscles were monitored to detect any startle-like response elicited by the perturbations^19,30^. Standard skin preparation techniques were used before applying single differential electrodes (DE-2.1; Delsys Inc., Natick, MA) to the skin. EMGs were amplified (Bagnoli^TM^-8 Desktop EMG system; Delsys Inc., Natick, MA), which has a bandwidth of 20–450 Hz. The amplified signals were anti-alias filtered at 500 Hz using custom fifth-order Bessel filters and then sampled at 2 kHz with an 18-bit analog-to-digital converter (NI PCI-6289; National Instruments, Austin, TX). A common clock was used to synchronize acquisition of endpoint displacements and forces from the robot with EMG sampling.

### Protocols

At the start of the experiment, a series of maximum voluntary contractions (MVCs) were performed using standard muscle testing procedures^31^. Separate isometric contraction was performed for each target muscle. Each contraction lasted for approximately 3 s and was performed twice. The MVCs recorded from these contractions were subsequently used to provide a relative measure of EMG activation for each subject.

**Figure 1:**
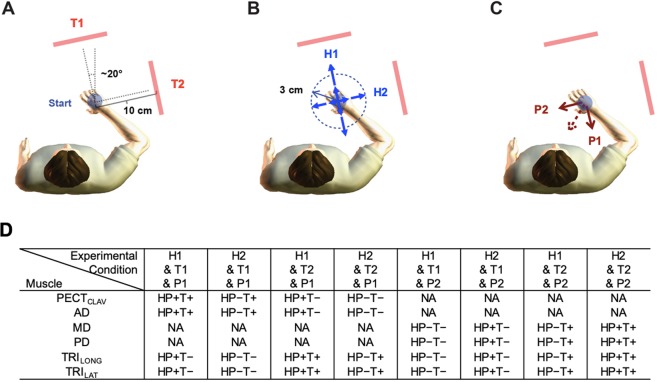
The schematic of the experiment. (**A**) Two final targets were used to independently manipulate target-dependent reactions; one outward in the direction of maximal stiffness (T1) and the other laterally along the second largest stiffness axis (T2). Both reaches were 10 cm. (**B**) Two haptic fields were implemented with a negative stiffness to challenge arm stability; one in T1 and the other in T2 directions (H1 and H2). For safety reason, unstable haptic fields were effective only when the hand was within 3 cm from the home position. (**C**) Two perturbations (P1 and P2, solid red arrows), opposite to T1 and T2 directions, were used to elicit stretch reflexes. A bias force of 5 N in the opposite direction of the middle of two targets (dotted red arrow) was used to reduce the variability in the elicited reflexes. (**D**) A summary of experimental conditions for each muscle. HP + (or HP−): orientation of the unstable haptic field was aligned (or not aligned) with direction of the perturbation. T+ (or T−): agonistic (or antagonistic) muscle action to a selected target. NA: not included in the analysis due to muscle shortening.

Our primary objective was to determine if stabilizing reflexes and target-dependent reactions could be modulated independently. This was accomplished using experimental protocols that independently accentuated these components of the rapid motor response elicited by an external perturbation of posture. The actions used in these experiments were ballistic reaches to two targets 10 cm from the home posture (Fig. 1A). We used two unstable haptic fields to modulate stabilizing reflexes of the rapid motor response (Fig. 1B): one (H1) oriented toward the first reaching target (T1) and another (H2) towards the second (T2). These unstable fields simulated a negative stiffness such that the robot pushed the hand away from the home position with a force proportional to the distance between the hand and the home position. Each field was unstable only in the specified direction; preferential increases in reflexes, elicited within 50–100 ms after perturbation onset, were expected when the orientation of the unstable haptic field was aligned with the direction of the perturbation. In addition to the simulated haptic stiffness, the robot was configured as a slightly under-damped 2^nd^ order system (damping ratio ζ = 0.5) with a simulated mass of 5.0 kg. For each subject, the magnitude of negative stiffness environments was selected to be large enough to challenge arm stability while maintaining comparable levels of muscle activity across both environments. As a result, the strength of the negative stiffness environment varied across subjects: −1800–−900 N/m for H1 and −800–−300 N/m for H2 (Table 1). The increased magnitudes for H1 resulted from the fact that this field orientation was selected to coincide approximately with the direction of maximum intrinsic arm stiffness^32^. To ensure safety of subjects, unstable fields were effective only when the hand was within 3 cm of the home position. Virtual walls (implemented with a simulated stiffness of 50 kN/m) were located at a distance of 5 cm from the home position.

**Table 1:** Strength of the negative stiffness values for unstable haptic environments.

Subject #	Sex	Field Strength, N/m	
		H1	H2
1	M	−1800	−800
2	M	−1200	−600
3	F	−1000	−400
4	M	−1500	−600
5	F	−1100	−500
6	M	−1100	−500
7	F	−1100	−500
8	M	−1100	−500
9	F	−900	−400
10	F	−900	−300

The main protocol involved having subjects reach ballistically from the home position to either T1 or T2, while interacting with either H1 or H2. Perturbations were used to elicit reflexes as subjects completed these tasks. Subjects worked against a constant bias force of 5 N (Fig. 1C) to reduce the variability of the perturbation-elicited reflexes. Perturbations were delivered in one of two directions, P1 (posterior direction) and P2 (medial direction), aligned with each of the two possible haptic fields (Fig. 1C). The condition where orientation of the unstable haptic field was aligned (or not aligned) with the perturbation direction or not was denoted as HP+ (or HP−). In addition, agonistic (or antagonistic) muscle action to a selected target was denoted as T+ (or T−). The alignment and muscle action conditions for each muscle were summarized (Fig. 1D). Haptic field and perturbation alignment and muscle action were used as two categorical independent variables in our statistical analysis.

Each perturbation had a speed of 350 mm/s and a duration of 100 ms to elicit consistent reflexes within the longer-latency period considered in this study^33^. These perturbations were delivered in random order, interspersed with a no perturbation (no P) condition to observe the quality of unimpeded reaches. This resulted in a total of three perturbation conditions (P1, P2, and no P), each delivered with equal probability. There were 20 repetitions of each experimental condition, resulting in 240 trials collected from each subject (2 reaching targets x 2 haptic fields x 3 perturbation conditions x 20 repetitions). Trials were presented in blocks of 12. The haptic field was kept constant within each block but randomized across blocks. This was done to help provide the subject with explicit information about the environment with which he/she was interacting. The remaining conditions were randomized and equally distributed within each block.

Prior to the start of each trial, subjects were instructed to move between two small targets, located at a distance of 1.5 cm from the home position along the direction of haptic field. Again, this was done to provide the subject with explicit information regarding the current haptic field. Each of these two targets was presented in a random order. After they were acquired, the home position appeared and the subject was instructed to place the hand at that location. Once in the home position, one of the two possible reaching targets (T1 or T2) was presented to the subject. After the home position was maintained for 1 s, a non-startling (80 dB) auditory WARNING cue was provided and subjects were instructed to prepare to reach as fast as possible towards the presented target. After a randomized time interval of 1–2 s, an auditory GO cue (80 dB) prompted subjects to initiate the reach. When perturbations were applied, they were presented concurrently with the GO cue. Each trial, including the field exploration, the postural hold period, the ballistic reach to the target, and the return to the home position, was completed in about 7 s. A minimum rest period of 2 minutes was provided between blocks. The total duration of the main session was about 70 minutes. A practice session of 36 trials, equally distributed across all 12 conditions, was used prior to the main experiment to familiarize subjects with this task. These practice data were not used in subsequent analyses.

### Analysis

Trials were excluded from the analysis if the subject failed to stay in the home position prior to the GO cue or failed to reach the specified target. This process led to the removal of 10.3 ± 1.8% (mean ± standard deviation (SD)) of the collected trials. EMG data were demeaned, rectified, and normalized by MVCs collected at the start of each experiment. The rapid motor responses elicited by the applied perturbations were quantified by the average response within three time windows relative to perturbation onset: 25–50 ms, 50–75 ms, and 75–100 ms, referred to as the short latency, early long-latency, and later long-latency, respectively. The short latency window has typically been used to characterize monosynaptic spinal reflexes, whereas the long latency windows have been used to estimate rapid feedback responses that can also include contributions from supraspinal pathways^14,19^.

The presence of SCM activity prior to 120 ms after perturbation onset was used to detect a startle-like response to the external perturbation^19,34,35^. Such responses have been associated with the rapid release of a planned motor response^19^. All trials in which the activity in either the left or right SCM exceeded 3 SD above the activity prior to the perturbation (100 ms window) were automatically flagged and then visually inspected to check for false positives. The reviewer was blinded to the trial type and the activity of other muscles during this inspection process. The perturbations used in this experiment consistently elicited activity in the SCM muscles; the probability of observing SCM activity in the perturbed trials was 0.89 ± 0.10. The average onset of SCM in these trials was 85.8 ± 5.1 ms. The probability of not observing SCM activity in the free reaching trials without perturbations was 0.86 ± 0.15. Trials with SCM activity were considered when analyzing the perturbation responses, and only trials without SCM activity were used for the free reaching movements in the absence of perturbations.

Our central hypothesis was that stabilizing reflexes and target-dependent reactions in stretched muscles can be modulated independently during the transition from posture to movement. This was assessed using linear mixed-effect models (*lme* function in *nlme* package in R) with haptic field and perturbation alignment (HP+ or HP−) and muscle action (T+ or T−) as fixed factors; subjects were treated as a random factor. Dependent variables were the average EMG response within the short latency, early long-latency, and later long-latency time windows. Separate analyses were performed for each muscle and time window. All trials were considered in the analysis to appropriately account for the variability associated with a different baseline for each subject, which decreases the probability of statistical error and thus has been shown to be more rigorous than using a single mean for each subject^36,37^. This analysis also ensures that data are not misrepresented due to unbalanced data sets (i.e., some subjects have fewer number of trials). Post-hoc pairwise comparisons were performed using the *contrast* package in R, which utilizes the Wald test to determine statistical significance of difference^38,39^. We expected the two main factors to be significant in long-latency windows. Considering the interactions between the two factors tested our central hypothesis that stabilizing reflexes and target-dependent reactions could be modulated independently. Lack of a significant interaction would provide evidence for independent modulation.

Our secondary hypothesis was that online corrections can rapidly modulate the time course of a target-dependent reactions. This was assessed by using linear mixed-effect models with target and perturbation directions as fixed factors and subjects as a random factor. Two arm muscles (TRI_LONG_ and TRI_LAT_) stretched by both posterior (P1) and medial (P2) perturbations were used in this analysis. This was assessed by investigating the interactions between target and perturbation directions in our statistical model. A significant interaction between target and perturbation directions would suggest that target-dependent reactions can be modified by online corrective processes.

## Results

**Figure 2:**
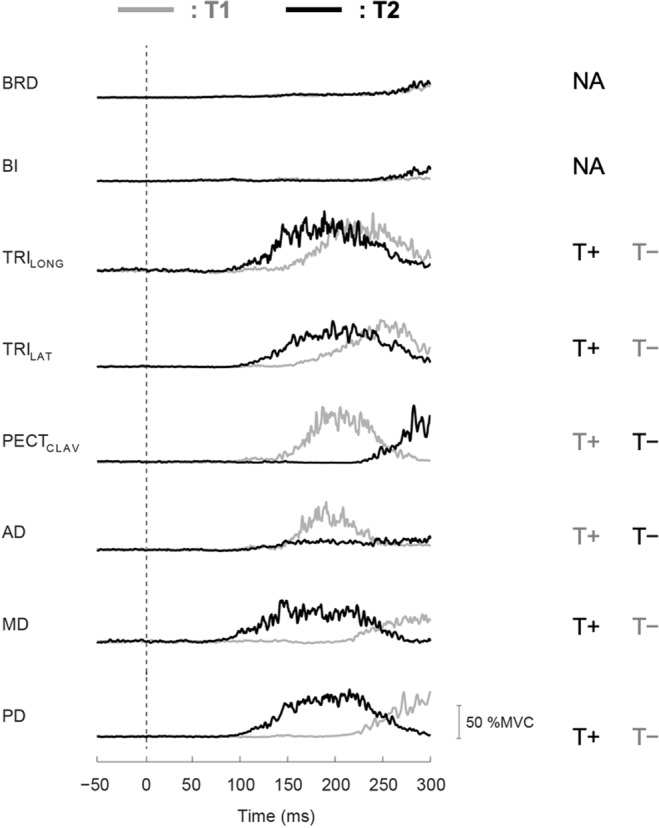
Typical average EMG responses of a representative subject during voluntary reaching. Normalized EMG responses (%MVC) during reaching to T1 (gray lines) and T2 (black lines). The GO cue occurred at 0 s. All muscles, except BRD and BI, had patterns of activity that differed between voluntary reaches to each of the targets. T+ or T−: target direction was aligned with agonistic muscle action or not. NA: no agonistic muscle action to both targets.

The recorded muscles had patterns of activity that differed between voluntary reaches to each of the targets used in these experiments, allowing the influence of target to be easily assessed in most muscle groups (Fig. 2). The shoulder flexors (AD and PECT_CLAV_) were activated early in reaches to T1, while the shoulder abductors and extensors (MD and PD) were activated early in reaches to T2. The elbow extensors (TRI_LONG_ and TRI_LAT_) were activated in less than ~170 ms during reaches to both targets, though earlier during reaches to T2 than T1. The elbow flexors (BRD and BI) were not strongly activated during reaches to either target and therefore were excluded from further analysis.

### Influence of environment and movement planning on perturbation-elicited reflexes

**Figure 3:**
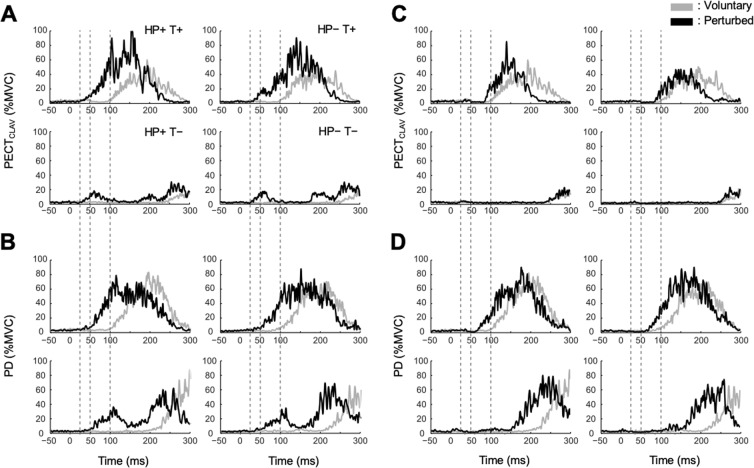
Typical average EMG responses of a representative subject during interaction with the environment challenging both postural stability and planned target reaching. HP+ (or HP−): the orientation of the unstable haptic field was aligned (or not aligned) with the direction of the perturbation. T+ (or T−): agonistic (or antagonistic) muscle action to the target. The following format applies to all figures: HP + T + (top left), HP−T+ (top right), HP+ T− (bottom left), HP−T− (bottom right). Normalized EMG responses (%MVC) with (black lines) and without perturbations (gray lines) were provided. Responses of PECT_CLAV_ and PD were provided as examples. (**A**,**B**) EMG responses when muscles were stretched. (**C**,**D**) EMG responses when muscles were shortened.

The rapid motor responses elicited by external perturbations of posture were modulated by the environment with which the subject was interacting. Average responses for a typical subject are shown in Fig. 3. The magnitude of the response elicited 50–100 ms after perturbation onset was greater when the orientation of the unstable haptic field was aligned with the direction of the perturbation (HP+) than when they were not aligned (HP−), a “field effect” that has been demonstrated previously^14^. For example, the EMG response in PECT_CLAV_ to P1, which stretched this muscle, was greatest when the subject interacted with the environment (H1) aligned with this perturbation direction (Fig. 3A). Similarly, the PD response to a stretch from P2 was greatest during interactions with H2 (Fig. 3B). While some modulation could be observed following perturbations that shortened the muscle, these were less consistent and confounded due to the fact that activity in shortened muscles was often strongly inhibited making it difficult to observe further modulation from surface EMG recordings (e.g. Fig. 3C,D). These responses were therefore excluded from further analysis since floor effects could contribute to false positive interactions in the linear modeling used for our analysis.

**Figure 4:**
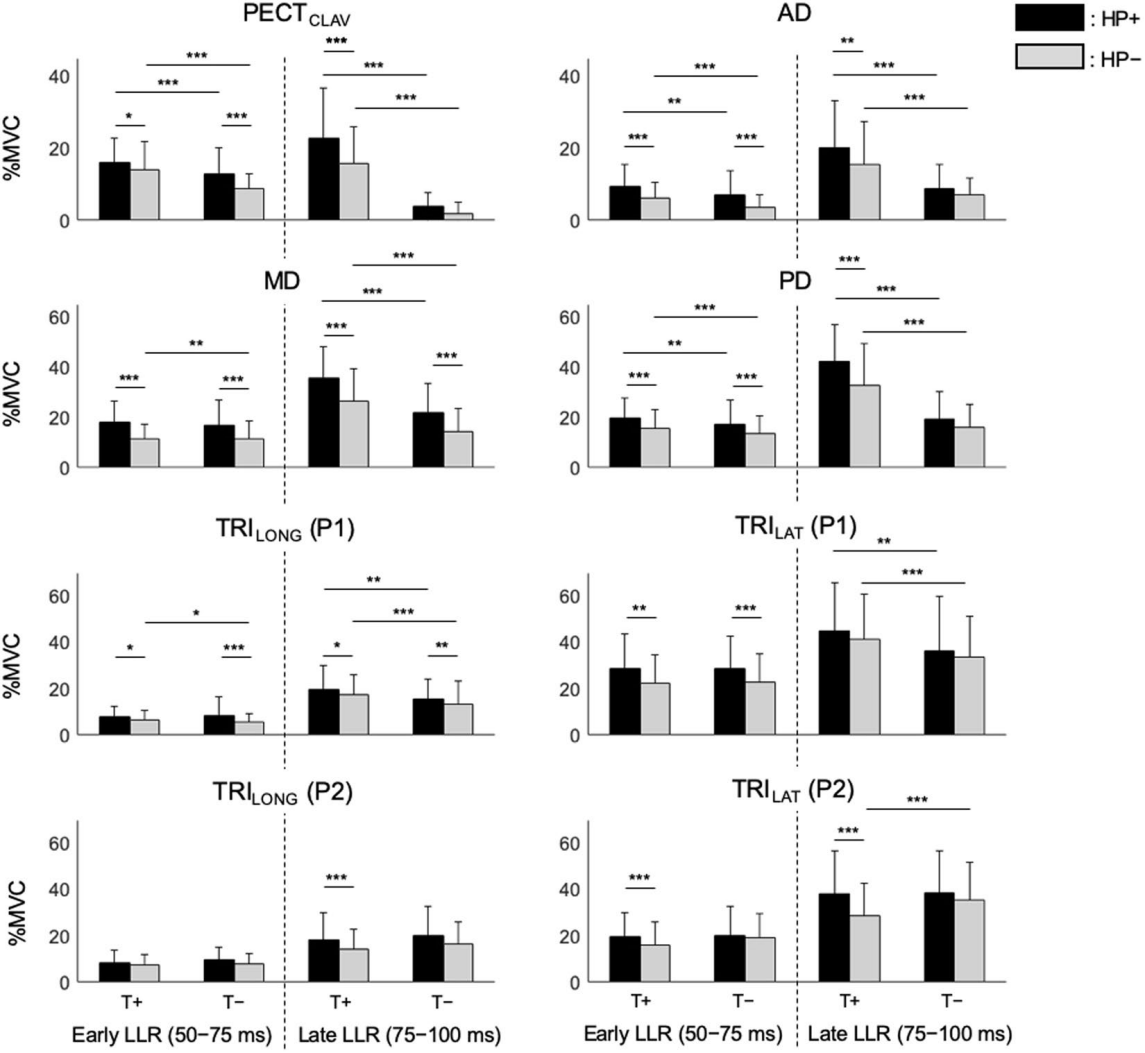
Group responses of reflex modulation for each combination of haptic field and perturbation alignment (HP+ or HP−) and muscle action (T+ or T−). The stretched reflexes elicited by the applied perturbations were quantified by the average normalized response (%MVC) within two long-latency time windows relative to perturbation onset: 50–75 ms and 75–100 ms, referred to as the early long-latency and later long-latency, respectively. Stars indicate significance in pairwise comparisons: *p < 0.05, **p < 0.01, ***p < 0.001. Error bars represent standard deviation (SD) after removing the random effect of subjects.

The field effect modulation for stretched muscles was consistent across subjects (Fig. 4) and was observed in both long-latency time windows (50–75 ms and 75–100 ms). When muscles were stretched by P1 (PECT_CLAV_, AD, TRI_LONG_, and TRI_LAT_), the elicited response was greater during interactions with H1 than H2. Similarly, the responses to muscles stretched by P2 (PD, MD, TRI_LONG_, and TRI_LAT_) were significantly greater during interactions with H2 than H1. This influence of the environment on the response to each perturbation resulted in significant field effect (∆HP in Table 2) in both long-latency windows in all muscles, except TRI_LAT_ to P1 in the later long-latency time window.

**Table 2:** Summary of statistical analysis.

Time	25–50 ms		50–75 ms		75–100 ms	
Muscle						
**∆HP**						
PECT_CLAV_	−0.28	—	3.45	***	1.67	***
AD	0.43	—	3.31	***	1.17	**
MD	1.50	—	5.82	***	7.33	***
PD	1.29	—	4.39	***	3.77	***
TRI_LONG_ (P1)	0.86	*	3.17	***	3.91	***
TRI_LONG_ (P2)	0.72	—	1.33	**	2.10	***
TRI_LAT_ (P1)	1.62	*	6.72	***	4.34	—
TRI_LAT_ (P2)	−1.38	—	0.74	*	2.30	***
**∆T**						
PECT_CLAV_	−0.21	—	4.31	***	13.64	***
AD	0.13	—	2.42	***	8.88	***
MD	1.14	—	3.02	**	13.24	***
PD	1.13	—	4.18	***	17.61	***
TRI_LONG_ (P1)	0.65	—	1.44	—	4.46	***
TRI_LONG_ (P2)	0.16	—	−0.002	—	−2.50	—
TRI_LAT_ (P1)	0.56	—	1.54	—	9.59	***
TRI_LAT_ (P2)	−1.02	—	−2.28	—	−7.89	*
∆**HP:T**						
PECT_CLAV_	0.73	—	−1.31	—	5.41	**
AD	0.21	—	−0.42	—	3.42	—
MD	−1.80	—	−1.45	—	0.55	—
PD	−0.78	—	−0.88	—	4.96	—
TRI_LONG_ (P1)	−0.55	—	−1.79	—	−1.05	—
TRI_LONG_ (P2)	−0.80	—	−0.14	—	2.35	—
TRI_LAT_ (P1)	0.07	—	−1.82	—	−1.70	—
TRI_LAT_ (P2)	0.09	—	2.90	—	8.21	*
**∆T:P**						
TRI_LONG_	−0.57	—	−0.50	—	−5.11	***
TRI_LAT_	−1.43	—	−1.60	—	−12.32	***

The amount of effect (∆) and statistical significance are presented (*p < 0.05, **p < 0.01, ***p < 0.001). The numerator DOF is 1 for all muscles. The denominator DOF for each muscle is as follow - PECT_CLAV_: 546, AD: 555, MD: 492, PD: 482, TRI_LONG_ (P1): 554, TRI_LONG_ (P2): 488, and TRI_LAT_ (P1): 556, and TRI_LAT_ (P2): 491.

The target towards which the subject had planned to reach also influenced the rapid response elicited by the perturbation (target effect). Overall, the magnitude of the response elicited 50–100 ms after perturbation onset was greater when the target direction was aligned with agonistic muscle action (T+) than when it was not (T−). In other words, greater responses reflected the activity of the muscle during unperturbed reaching to each target (Fig. 2). This observation occurred regardless of the direction of the unstable haptic field. For example, the PECT_CLAV_ response was greater when planning to reach to T1 than to T2 in both haptic field conditions (Fig. 3A). Similarly, the PD response was greatest when planning to reach to T2 (Fig. 3B).

For the subjects in this study, the target effect was consistent for PECT_CLAV_, AD, MD, and PD in both long-latency time windows (Fig. 4; ∆T in Table 2). The target effect was always largest in the later time window (75–100 ms) for these four muscles; the magnitude of the response in the later window was 3.9 ± 0.6 (mean ± SD) times greater than that in the earlier window (50–75 ms). TRI_LONG_ and TRI_LAT_ were observed to have statistically insignificant target effects in the earlier long-latency time window, possibly since the target-dependent difference in the onset times of these muscles was smaller during voluntary reaches than the other muscles considered for analysis (Fig. 2). In the later long-latency time window, both triceps muscles showed the significant positive target effect to P1.

The field effect and the target effect were independent during the early long-latency window (50–75 ms). Within this period the modeled interaction terms did not reach statistical significance for any muscles (∆HP:T in Table 2). In the later long-latency window, all muscles except PECT_CLAV_ and TRI_LAT_ responses to P2 did not reach statistical significance. The significant interaction in PECT_CLAV_ and TRI_LAT_ responses to P2 can be attributed to the larger field effect during reaching to T+ than T− (Fig. 4). This trend was also observed in AD and PD but did not reach statistical significance (*p* = 0.09 and 0.07 for AD and PD, respectively).

The shorter latency responses elicited 25–50 ms after perturbation onset were small relative to the longer latency responses, and not strongly influenced by either the orientation of the haptic field or the target towards which the subject was reaching (Table 2). Only triceps responses to P1 showed significant environment specific modulation, but it was substantially smaller than that in long-latency windows. There was no significant interaction between the field effect and the target effect for any muscles in the short-latency time window.

### Online corrections contribute to rapid target-dependent reactions

**Figure 5:**
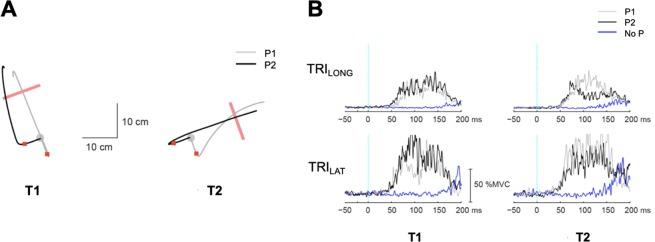
Target-dependent reactions of a representative subject for different directions of the perturbation. (**A**) Kinematic responses for each combination of target direction and perturbation direction. Red bars, gray circles represent targets and the home position. The red square denotes the hand position 100 ms after perturbation onset. (**B**) Different EMG responses for different perturbation conditions, i.e., P1 (posterior perturbation) and P2 (medial perturbation) for each target.

The rapid target-dependent reactions exhibited clear dependence on the direction of the perturbation used to elicit the response, suggesting that the involuntary release of the planned motor response is not a pure triggered reaction (i.e., motor output is preprogramed and released in response to the perturbation) but can be modified by online corrective processes. The EMG response in the long-latency time window in TRI_LONG_ and TRI_LAT_ was greater to P2 when the subject planned to reach to T1, while the response was greater to P1 when planned to reach to T2 (Fig. 5B). This corrective response was consistent in the later long-latency time window (75–100 ms) across subjects (Table 2). This was assessed by examining the interaction term between target direction and perturbation direction in our statistical model (∆T:P; Table 2). While the interaction was significant in the later long-latency time window, it was not in the short and early long-latency time windows.

## Discussion

Interaction with the physical world is one of our most basic needs, requiring seamless integration of involuntary and voluntary control mechanisms. Rapid motor responses to external perturbations of posture play an important role at the interface between involuntary and volitional control. These responses can be complex, adapting to facilitate interactions with different mechanical environments, movement goals, or other features pertinent to the successful control of movement and posture. The purpose of this study was to examine interactions between distinct behaviors within the rapid motor responses elicited in the time period commonly ascribed to LLR. Specifically, we were interested in the interactions between stabilizing components of the LLR and the rapid release of planned movements, both in elicited response to external perturbations of posture. We found that rapid responses were modulated by the environment with which the subject was interacting (field effect) and the target direction to which the subject was planning to reach (target effect), but that there was little interaction between these effects particularly in the early portion of the LLR (50–75 ms). This result suggests that stabilizing reflexes and target-dependent components of the LLR can be modulated independently. Importantly, we observed that the target effect can be modulated by the direction of the postural perturbation applied during a reach in the later period of the LLR (75–100 ms), suggestive of later online corrections after an initial release of a planned response. These results demonstrate how distinct components of the long-latency reflex can work independently and together to generate sophisticated rapid motor responses that integrate planning with reaction to uncertain conditions.

Our results are consistent with the growing consensus that multiple convergent pathways contribute to perturbation-evoked motor responses in time period often attributed to the LLR^16,25–27^. Several studies have suggested that the stabilizing components of the LLR are at least partially mediated by motor cortical pathways. Recordings from pyramidal tract neurons in non-human primates showed that transcortical reflexes are more relevant to the precise control of posture than to the generation of ballistic movements triggered by an external perturbation^40^. Results from non-human primates and human subjects have demonstrated cortical involvement in the LLR responses coordinating inter-joint responses to external perturbations. Transcranial magnetic stimulation (TMS) has also been used in human subjects to demonstrate that modulation of the LLR due to changes in the haptic environment, including those similar the experiments presented here, can be blocked through a temporary silencing of cortical activity^41,42^.

There is less consensus on the pathways contributing to LLR modulation coupled to motor planning. Many studies suggest involvement of subcortical structures. In non-human primates, neurons in the pontomedullary reticular formation exhibit movement-related and preparatory activity during rapid planar reaching^43^. Rapid triggering of planned movements can be elicited in humans using startling acoustic stimuli (SAS), known to activate brainstem pathways^44–46^. Similar rapid responses, including electrophysiological signatures of brainstem activity, can also be elicited by postural perturbations suggesting that mechanically triggered responses may also involve the brainstem pathways^19^. These conclusions from studies on unimpaired subjects are consistent with recent results from stroke survivors^47–49^, patients with pure hereditary spastic paraplegia^50^, and spinal cord injury patients^51^, all demonstrating SAS-triggered motor responses even in the presence of an impaired corticospinal system. Alternatively, some have implicated the motor cortex in the release of SAS and perturbation-triggered reactions. These conclusions have been based in part on the observations that perturbations can trigger rapid responses reflecting a planned movement even in the absence of electrophysiological indicators of brainstem activity^52^, and that the motor activity elicited by SAS is additive with cortically mediated volitional activation^53^. Finally, it is quite plausible that cortical and subcortical circuits are involved in many paradigms used to study the rapid release of planned movements. There is strong neuroanatomical evidence demonstrating significant cortical projections to brainstem pathways^54,55^. There is also experimental evidence that cortical silence from TMS significantly delays the effects of SAS on the rapid release of a planned motor action^56,57^. Regardless of the neural origin for perturbation-triggered reactions, our results demonstrate that their earliest manifestations can be modulated independently from the stabilizing actions of the LLR. This allows for a rapid motor response that can simultaneously be tuned for multiple objectives: stability and rapid movement.

While our results demonstrated a clear component of the early LLR that was dependent on the planned movement, the later LLR was also modulated by the direction of the applied perturbation. This later modulation was appropriate for correcting perturbation-induced errors in the movement trajectory, reflective of feedback control. These results are consistent with multiple studies demonstrating continuous online corrections when making rapid movements to targets^17,22,24,28,58,59^. We believe they also demonstrate that triggered reactions are not inconsistent with the concepts of feedback control but rather that both behaviors can contribute to the response to a perturbation. The precise expression depends in part on the conditions prior to the perturbation and on how the subject is instructed to respond.

Together our results demonstrate how three important components of the LLR interact during a task with multiple goals. In the earliest portion of the LLR, the stabilizing components of the reflex that resist external perturbations of posture are modulated independently from the rapidly triggered motor responses reflecting a movement planned prior to the perturbation. The later portion of the response also can reflect online corrections as needed to complete the task given to the subject. The complexity of this response and the multiple components contributing to it demonstrate the rich repertoire of motor responses that are available for rapid deployment by the central nervous system.

## Data Availability

The data sets generated during and/or analyzed during the current study are available from the corresponding author on reasonable request.
